# Resveratrol attenuates intestinal epithelial barrier dysfunction via Nrf2/HO‐1 pathway in dextran sulfate sodium‐induced Caco‐2 cells

**DOI:** 10.1002/iid3.1193

**Published:** 2024-02-19

**Authors:** Xinya Yu, Yazhi Wang, Yunchun Xu, Xiaoxi Li, Junhua Zhang, Yunpeng Su, Le Guo

**Affiliations:** ^1^ Department of Medical Microbiology and Immunology, School of Basic Medical Sciences Dali University Dali Yunnan People's Republic of China; ^2^ Department of General Surgery, School of Clinical Medicine Dali University Dali Yunnan People's Republic of China

**Keywords:** intestinal barrier function, intestinal epithelial cells, Nrf2/HO‐1, resveratrol, tight junction

## Abstract

**Introduction:**

The intestinal tract serves as an innate barrier, safeguarding the internal milieu from microorganisms and toxins. Various intestinal inflammatory diseases have a strong association with intestinal barrier dysfunction. The primary functional cells within the intestinal tract, intestinal epithelial cells (IECs) and their tight junctions (TJs), are crucial in preserving the integrity of this mechanical barrier. Resveratrol (Res), a plant‐derived phenolic compound, exhibits a range of health‐promoting benefits attributed to its anti‐inflammatory properties. This study aims to examine Res's efficacy in bolstering IECs barrier function.

**Methods:**

Dextran sulfate sodium (DSS) was employed to induce barrier dysfunction in IECs. Inflammatory cytokines in supernatants (interleukin [IL]‐6, IL‐1β, tumor necrotic factor [TNF]‐α, and IL‐10) were quantified via enzyme‐linked immunosorbent assay (ELISA). Then we assessed monolayer integrity using transepithelial electrical resistance (TEER). TJ protein expression (zonula occludens [ZO]‐1 and Occludin) in IECs was evaluated through immunofluorescence and Western blot analysis. Network pharmacology helped identify the biological processes, signaling pathways, and key targets involved in Res's mitigation of DSS‐induced IECs barrier dysfunction. The efficacy of the primary target was further corroborated using Western blot.

**Results:**

Res was shown to increase cell viability and IL‐10 expression while reducing TNF‐α, IL‐6, and IL‐1β levels, thus mitigating the inflammatory response. It enhanced TEER values and upregulated TJ protein expression (ZO‐1 and Occludin). Network pharmacology revealed that Res potentially targets the NFE2L2 (nuclear factor erythroid‐2‐related factor 2, Nrf2), a vital antioxidant factor. Significantly, Res augmented Nrf2 and heme oxygenase 1 (HO‐1) protein levels, counteracting oxidative stress in the IECs barrier dysfunction model.

**Conclusion:**

Overall, our findings suggested that Res ameliorated DSS‐induced IECs barrier dysfunction by activating Nrf2/HO‐1 pathway, showcasing significant therapeutic potential in the early stages of colitis.

## Graphical abstract

Our findings indicated that Res attenuated dextran sulfate sodium‐induced intestinal epithelial cells barrier dysfunction via activating nuclear factor erythroid‐2‐related factor 2/heme oxygenase 1 (Nrf2/HO‐1)pathway and had great therapeutic potential throughout the preliminary stages of colitis.

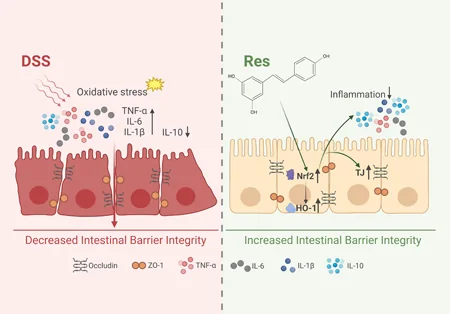

## 1 INTRODUCTION

The intestinal mucosa serves as a selective permeable barrier, critical for nutrient digestion and absorption, and shields the internal environment from toxins and pathogenic bacteria. The human intestinal tract harbors a multitude of microorganisms, including bacteria, viruses, fungi, and helminths, collectively known as the “intestinal microbiota.”^1^ This microbiota and the intestinal barrier engage in a complex, balanced interaction network under normal physiological conditions, fostering human homeostasis and health.^2^ The intestinal barrier is multilayered: the outer layer comprises the mucus layer, symbiotic intestinal microflora, and defensive proteins such as antimicrobial proteins and secretory immunoglobulin A. The middle layer consists of intestinal epithelial cells (IECs), and the innermost layer is made up of innate and adaptive immune cells.^3^

IECs, the body's fastest‐renewing cells, act as intermediaries between the internal and external environments of the intestinal tract. Together with tight junctions (TJs) between the cells, they form the intestinal mechanical barrier. The formation and assembly of TJs are involved in a variety of proteins, among which the cytoplasmic protein zonula occludens (ZO)‐1 and the transmembrane protein Occludin play a vital role in maintaining the TJ of IECs and improving intestinal permeability. In a state of homeostasis, TJs occlude the paracellular space between adjacent IECs, preventing bacteria and antigens from penetrating the lamina propria of the intestinal mucosa, thereby averting abnormal mucosal immune responses.^4^ IECs barrier dysfunction is linked to various diseases, ranging from autoimmune disorders (such as inflammatory bowel disease [IBD], type 1 diabetes, and systemic lupus erythematosus) to neurological conditions (including Parkinson's and Alzheimer's diseases).^5^, ^6^ Given the crucial role of IEC barrier regulation in health, significant attention has been directed toward the involvement of TJs in disease prevention and treatment.

Previous studies have established that dietary polyphenols, such as naringin and quercetin, enhanced the expression of TJ proteins, thereby maintaining the integrity of the IECs barrier and reducing inflammation induced by lipopolysaccharide (LPS).^7^, ^8^ However, the specific role of polyphenols in IECs barrier function remains under‐explored. Resveratrol (Res), a naturally occurring polyphenol found in grapes, blueberries, *Polygonum cuspidatum*, and peanuts, modulates various cellular targets and signaling pathways, including nuclear factor erythroid‐2‐related factor 2 (Nrf2), nuclear factor kappa‐B (NF‐κB), and adenosine 5'‐monophosphate (AMP)‐activated protein kinase. It is predominantly associated with neuroprotection, antioxidant activity, and inflammation modulation.^9^, ^10^, ^11^, ^12^ Res has demonstrated efficacy in mitigating diseases such as cancer, coronary heart disease, diabetes, and neurodegenerative disorders like Parkinson's and Alzheimer's disease.^13^, ^14^ It has also been reported to inhibit the progression of colon cancer and ulcerative colitis (UC) in mice.^15^, ^16^ Although various mechanisms contribute to the onset and progression of these diseases, recent findings suggested that IECs barrier dysfunction played a role in their pathogenesis. Therefore, the regulation of the IECs barrier could be a key mechanism in Res's therapeutic effects on these diseases.

Nrf2 is the primary regulator of the in vivo antioxidant defense system and is involved in signal transduction related to various intracellular defense mechanisms.^17^ Under normal physiological conditions, Nrf2 and Kelch‐like ECH‐associated protein‐1 form an inactive complex in the cytoplasm. In response to oxidative stress and elevated reactive oxygen species (ROS), this complex dissociates, allowing Nrf2 to enter the nucleus and regulate the gene expression of detoxification enzymes and antioxidant proteins via the antioxidant response element. This process enhances cellular detoxification and antioxidant capacity.^18^ Heme oxygenase 1 (HO‐1), a downstream target protein of Nrf2, decomposes heme to release biliverdin, CO, and ferrous ion (Fe^2+^). The Nrf2/HO‐1 pathway exhibits numerous functions, including antioxidant, anti‐inflammatory activities, maintenance of mitochondrial homeostasis, apoptosis prevention, and cell death regulation. Research indicates that the gastrointestinal tract is a major source of ROS, and Nrf2 has been shown to mitigate intestinal mucosal injury by reducing oxidative stress, inhibiting inflammatory pathways, decreasing intestinal inflammation, and regulating intestinal permeability, thus playing a protective role in maintaining the intestinal barrier.^19^

Remarkably, Res can modulate the Nrf2/HO‐1 pathway, combating a range of diseases such as enhancing the antioxidant capacity in an Alzheimer's disease mouse model and reducing acute lung injury in septic rats by inhibiting inflammation, oxidative stress, and apoptosis.^20^, ^21^ Consequently, it is hypothesized that Res might act as a novel adjuvant therapy, regulating the intestinal barrier and protecting IECs from injury via the Nrf2/HO‐1 pathway. Thus, this study aimed to develop a dextran sulfate sodium (DSS)‐induced IECs barrier dysfunction model at cellular and molecular levels to investigate the protective effects of Res and mechanisms on IECs barrier dysfunction, providing a theoretical and experimental foundation for treating intestinal diseases.

## 2 MATERIALS AND METHODS

### 2.1 Cell culture

The human colon cancer epithelial cell line (Caco‐2) (Procell) was cultured in Dulbecco's modified eagle's medium (Gibco), supplemented with 20% fetal bovine serum (Gibco), at 37°C in a humidified atmosphere of 5% CO_2_.

### 2.2 Cell viability assay

IECs were seeded in 96‐well plates at a density of 4 × 10^4^/well. Once fully differentiated and at 80%–90% confluency, cells were treated with Res (purity ≥98%, Solarbio) at concentrations of 5, 10, 20, 30, 40, 50 µM for 24 h, or pretreated with designated concentrations of Res for 1 h, followed by 2.5% DSS (Acmec) incubation for 24 h. Subsequently, 10 µL cell‐counting kit‐8 (CCK‐8) solution (Biosharp) was added to each well and incubated for an additional 30 min. The absorbance was then measured at a wavelength of 450 nm.

### 2.3 Measurement of transepithelial electrical resistance (TEER)

IECs were seeded in Transwell chamber (6.5 mm diameter, 8.0 μm pore size; Corning, NY, USA) at a density of 4 × 10^4 ^cells/insert (0.33 cm^2^) and cultured for 21 days until complete differentiation. The medium was routinely refreshed every other day, and monolayer integrity was assessed by measuring TEER with a Millicell ERS‐2 epithelial cell volt‐ohm meter (Merck Millipore). To evaluate Res's effect against DSS‐induced IECs monolayer injury, after 1 h incubation with 5, 10, 20 µM Res, 2.5% DSS was added, and resistance was measured at intervals of 6, 12, 24, 36, 48 h posttreatment, calculated using Equation (1).

(1) $$\mathrm{TEER}\left(\Omega ∙{\mathrm{cm}}^{2}\right)=(\mathrm{cells}\mathrm{resistance}-\mathrm{blank}\mathrm{resistance})\times 0.33{\mathrm{cm}}^{2}.$$

### 2.4 Enzyme‐linked immunosorbent assay (ELISA)

Cell‐free supernatants were collected to measure cytokine protein levels secreted by IECs. The expression of tumor necrotic factor (TNF)‐α, IL‐6, IL‐1β, and IL‐10 was determined using ELISA kits (4A Biotech), adhering to the manufacturer's protocols. Each well's absorbance was measured at a wavelength of 450 nm using a microplate reader.

### 2.5 Western blot analysis

Total proteins from IECs were extracted using RIPA buffer (Beyotime). Protein concentrations were quantified using the enhanced bicinchoninic acid Protein Assay kit (Beyotime). Following sodium dodecyl sulfate polyacrylamide gel electrophoresis separation, proteins were transferred to a polyvinylidene difluoride membrane (Merck Millipore), blocked with 5% nonfat milk for 2 h, and incubated with primary antibodies targeting ZO‐1, Occludin, Nrf2, HO‐1 and glyceraldehyde‐3‐phosphate dehydrogenase (GAPDH) (1:1000 dilution, Cell Signaling Technology) overnight at 4°C. The following day, the membranes were incubated with secondary hprseradish peroxidase‐conjugated goat antirabbit IgG (1:5000 dilution, Cell Signaling Technology) at room temperature for 1 h. GAPDH was used as a loading control. Signals were detected using BeyoECL Moon (Beyotime) and chemiluminescence imaging systems (Cytiva, ImageQuant LAS 4000 mini) and analyzed with Image J.

### 2.6 Immunofluorescence

IECs were rinsed with phosphate‐buffered saline (PBS), fixed in precooled 4% paraformaldehyde for 30 min, and blocked with 1% bovine serum albumin at room temperature for 1 h. They were then incubated with primary antibodies against ZO‐1 (1:200 dilution, Cell Signaling Technology) and Occludin (1:300 dilution, Cell Signaling Technology) overnight at 4°C. The following day, cells were treated with secondary fluorescein isothiocyanate‐conjugated goat antirabbit IgG (1:200 dilution, Cell Signaling Technology) in darkness for 1 h. IECs were subsequently stained with 4'‐6‐diamidino‐2‐phenylindole for nuclear visualization and washed thrice with PBS. Images were captured using an Olympus FLUOVIEW FV1000 Microscope.

### 2.7 Network pharmacology analysis

Initially, the Traditional Chinese Medicine Systems Pharmacology (TCMSP) database (http://tcmspw.com/tcmsp.php) was utilized to identify Res‐related targets, and the UniProt database^22^ (https://www.uniprot.org) was employed for converting these target proteins into corresponding human genes. Concurrently, genes associated with colitis were sourced from the GeneCards database (https://www.genecards.org). The online tool Venny2.1^23^ (https://bioinfogp.cnb.csic.es/tools/venny) facilitated the identification of overlaps between Res targets and colitis‐related genes. Further analysis of these overlapping targets was conducted using the DAVID^24^ (https://david.ncifcrf.gov/home.jsp) and Kyoto Encyclopedia of Genes and Genomes (KEGG) pathway^25^ (https://www/genome.jp/kegg/pathway.html) databases for Gene Ontology (GO) function enrichment and KEGG pathway analysis. Additionally, potential compound‐disease target genes were imported into the STRING database^26^ (https://string-db.org) for protein–protein interaction (PPI) analysis. The Cytoscape application visualized the interaction network. The selected hub gene, associated with anti‐inflammatory and antioxidant properties, was chosen for further in vitro validation.

### 2.8 Small‐interfering RNA (siRNA) transfection

The sequences of Nrf2 siRNA (General Biol) were as follows: sense strand 5′‐UGACAGAAGUUGACAAUUATT‐3′. Nrf2 and negative control siRNA were transfected according to the manufacturer's instructions using Lipo8000™ Transfection Reagent (Beyotime).

### 2.9 Statistical analysis

All experiments were performed in triplicate. Data are presented as mean ± SD. Statistical analyses were conducted using the *t* test and one‐way analysis of variance with GraphPad Prism version 9.0. *p *< .05 was considered regarded as statistically significant.

## 3 RESULTS

### 3.1 Res increases IECs viability and alleviates inflammation induced by DSS

**Figure 1:**
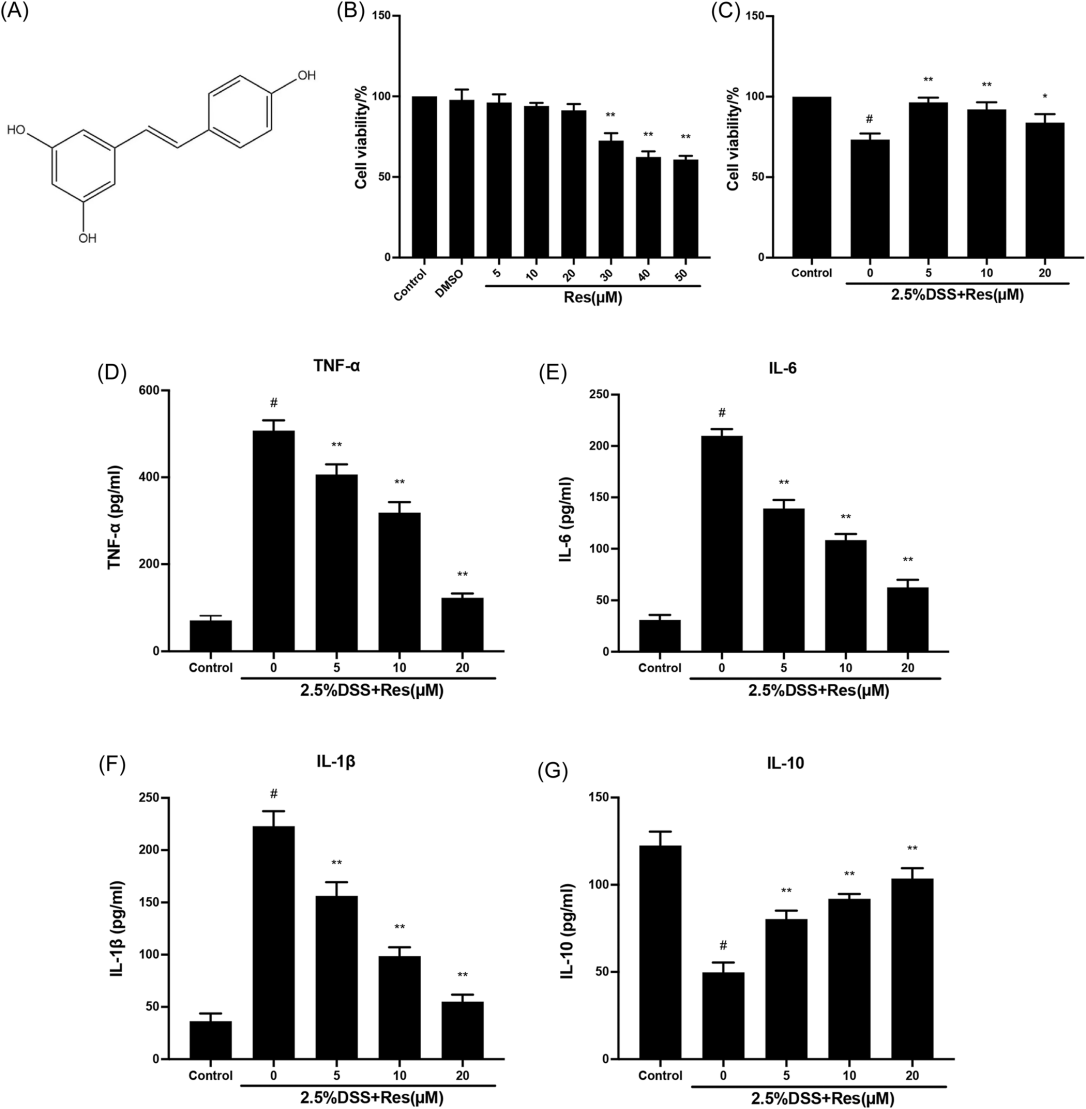
Resveratrol (Res) increases intestinal epithelial cells (IECs) viability and alleviates inflammatory response induced by dextran sulfate sodium (DSS). (A) Chemical structure of Res. (B) Cell viability when IECs were treated with the indicated concentrations of Res for 24 h. (C) Cell viability when IECs were pretreated with the indicated concentrations of Res for 1 h and then stimulated with 2.5%DSS for 24 h. (D–G) The effect of Res on proinflammatory cytokines (tumor necrotic factor [TNF]‐α, interleukin [IL]‐6, and IL‐1β) and anti‐inflammatory cytokine (IL‐10) expression were determined by enzyme‐linked immunosorbent assay (ELISA). ^#^ *p* < .01 versus control group; *p < .05, **p < .01 versus DSS group.

Figure 1A illustrates the chemical structure of Res. IECs were treated with the concentrations of Res (5, 10, 20, 30, 40, and 50 µM) for 24 h to evaluate its cytotoxic effects. Cell viability was determined using the CCK‐8 assay. As depicted in Figure 1B, Res concentrations of 5–20 µM had no toxic effects on IECs compared to the control group, whereas concentrations of 30–50 µM significantly reduced cell viability. Consequently, Res doses of 5, 10, and 20 µM were used in subsequent experiments. The effect of Res pretreatment at these concentrations on IECs viability in the presence of DSS was then evaluated. Compared to the DSS group, Res pretreatment markedly enhanced cell proliferation (Figure 1C).

It is established that TNF‐α, IL‐6, and IL‐1β are key proinflammatory cytokines involved in the onset of colitis,^27^ and IL‐10, an anti‐inflammatory cytokine, increases following Res administration in IBD patients.^28^ Our findings revealed that DSS upregulated TNF‐α, IL‐6, and IL‐1β (Figure 1D–F) and downregulated IL‐10 (Figure 1G). However, Res counteracted these effects in a dose‐dependent manner. In summary, Res demonstrated the potential to attenuate DSS‐induced IECs inflammatory response at the protein level.

### 3.2 Res enhances IECs barrier integrity and TJ proteins expression

**Figure 2:**
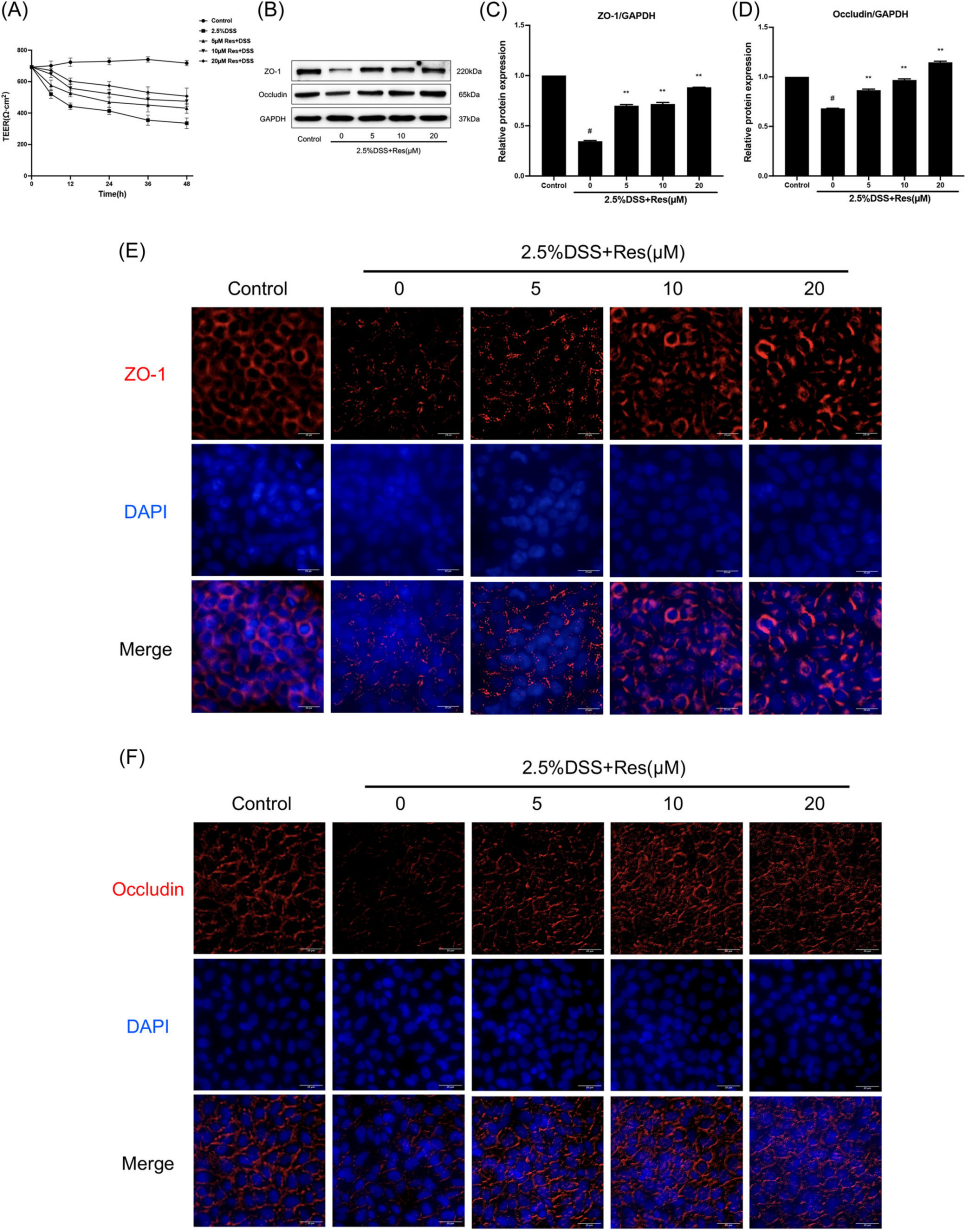
Resveratrol (Res) enhances intestinal epithelial cells (IECs) barrier integrity and tight junction (TJ) proteins expression. (A) Regulatory effect of Res on cell monolayer transepithelial electrical resistance (TEER) value. (B–D) Respectively Western blot and quantification data of TJ proteins (zonula occludens [ZO]‐1 and Occludin). (E and F) Immunofluorescence assessed ZO‐1 and Occludin distribution. IECs were pretreated with the indicated concentrations of Res for 1 h and then stimulated with 2.5% dextran sulfate sodium (DSS) for 24 h. Scale bar, 20 µM. ^#^ *p* < .01 versus control group; **p < .01 versus DSS group.

TEER, a classical biological index, evaluates the monolayer integrity of IECs. Treatment with DSS significantly reduced TEER values, showing a 51.63% decrease at 48 h. In contrast, Res‐treated groups exhibited higher resistance values than the DSS group. Specifically, after 48 h of DSS induction, the resistance in the 5–20 µM Res groups decreased by 37.83%, 31.39%, and 24.08%, respectively (Figure 2A), suggesting that Res pretreatment could enhance the monolayer integrity and barrier function of IECs.

DSS suppressed the expression of TJ proteins, whereas Res at concentrations of 5–20 µM increased the expression of ZO‐1 and Occludin to varying degrees (Figure 2B–D). Immunofluorescence analysis corroborated these findings for ZO‐1 and Occludin, aligning with the Western blot results (Figure 2E,F). Overall, these results indicated that Res is capable of maintaining the barrier integrity of IECs.

### 3.3 NFE2L2 serves as a potential target of Res in colitis relief

**Figure 3:**
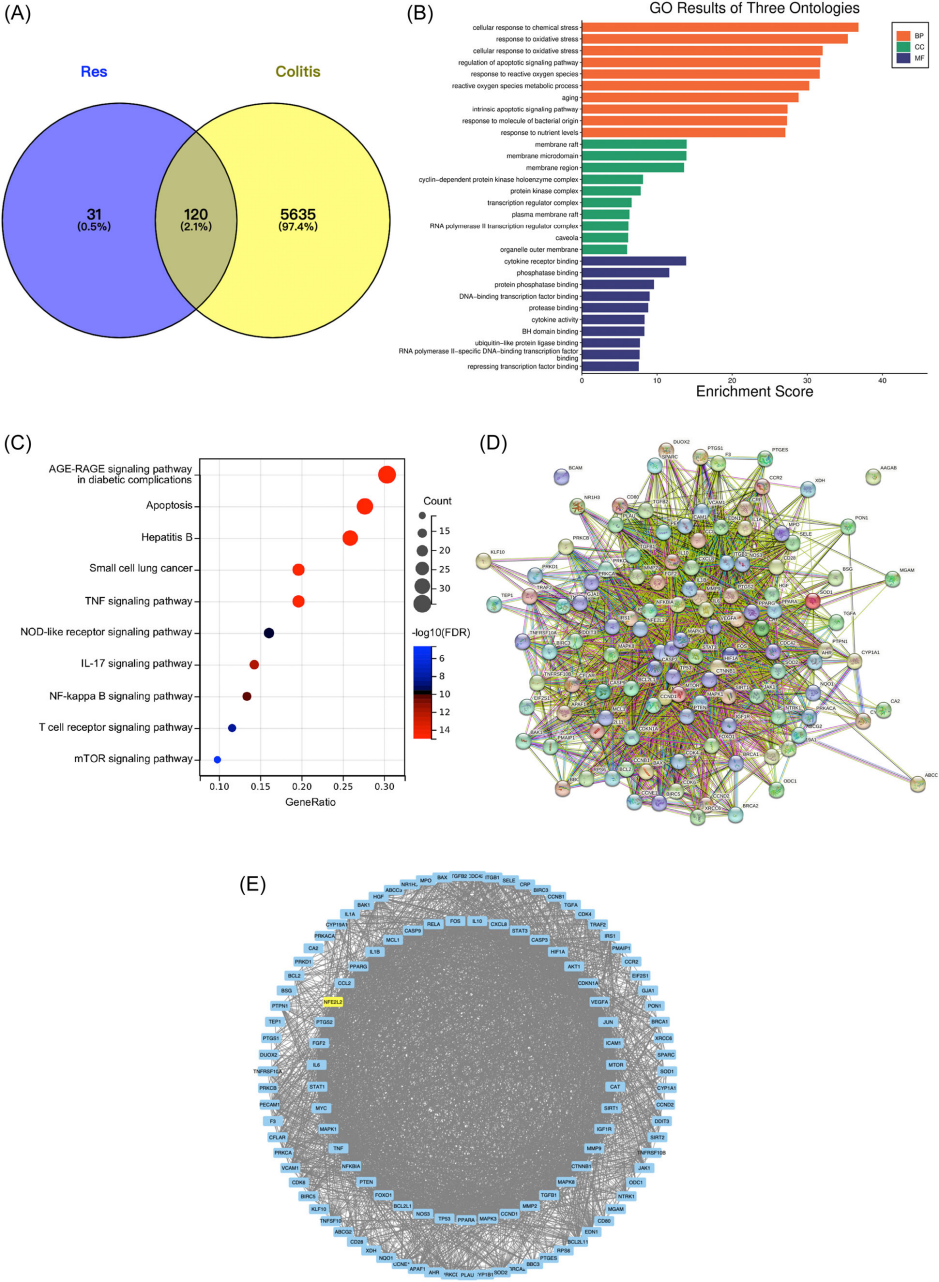
Nuclear factor erythroid‐2‐related factor 2 serves as a potential target of resveratrol (Res) in relieving colitis. (A) The overlapping genes between Res targets and colitis targets were examined by Venny2.1. (B) Analysis of Gene Ontology (GO) enrichment of overlapping genes between compound targets and disease targets. (C) Analysis of Kyoto Encyclopedia of Genes and Genomes (KEGG) pathway of overlapping genes between compound targets and disease targets. (D) Protein–protein interaction (PPI) networks of Res for the treatment of colitis. (E) Cytoscape mapping results by degree values.

Our findings demonstrated that Res could mitigate DSS‐induced IECs barrier dysfunction, but the underlying mechanism remained elusive. Consequently, we identified Res's potential targets that may regulate colitis using network pharmacology analysis. Initially, 151 Res target proteins were identified from TCMSP and backtranslated into corresponding genes using UniProt. Subsequently, employing the term “colitis,” we retrieved 5755 related genes from the GeneCards database. Venny2.1 identified 120 overlapping genes between compound targets and disease‐related genes (Figure 3A).

GO enrichment and KEGG pathway analysis of the 120 targets clarified Res's function and pharmacological mechanism. The analysis covered biological process (BP), cell component (CC), and molecular function (MF) terms for GO enrichment. As shown in Figure 3B, the top 10 enriched items in BP, CC, and MF terms were revealed to be an introduction to the GO enrichment. BP and MF highlighted the involvement in cytokine receptors, phosphatase activity, and DNA interactions with chemical stress, oxidative stress, apoptosis, and ROS. Membrane raft, membrane microdomain, and membrane region were the key components of CC. KEGG analysis suggested that pathways like apoptosis, TNF, nucleotide oligomerization domain‐like receptor, IL‐17, and NF‐kappa B signaling pathways might be crucial in regulating colitis (Figure 3C).

We then utilized the STRING database to establish PPI connections among the 120 target genes. Selecting medium confidence target proteins with interaction scores >0.4, we constructed a PPI network (Figure 3D), which was visualized using Cytoscape. Among these genes, we found that NFE2L2, which was previously noticed by our research group, was also included, and the correlation was high. In addition, NFE2L2 is involved in the anti‐inflammatory and antioxidant pathways both outside and inside the cell. Therefore, we hypothesized that Res might protect IECs by modulating the anti‐inflammatory and antioxidant pathways involving NFE2L2 (Figure 3E).

### 3.4 Res activates Nrf2/HO‐1 pathway in DSS‐induced IECs

**Figure 4:**
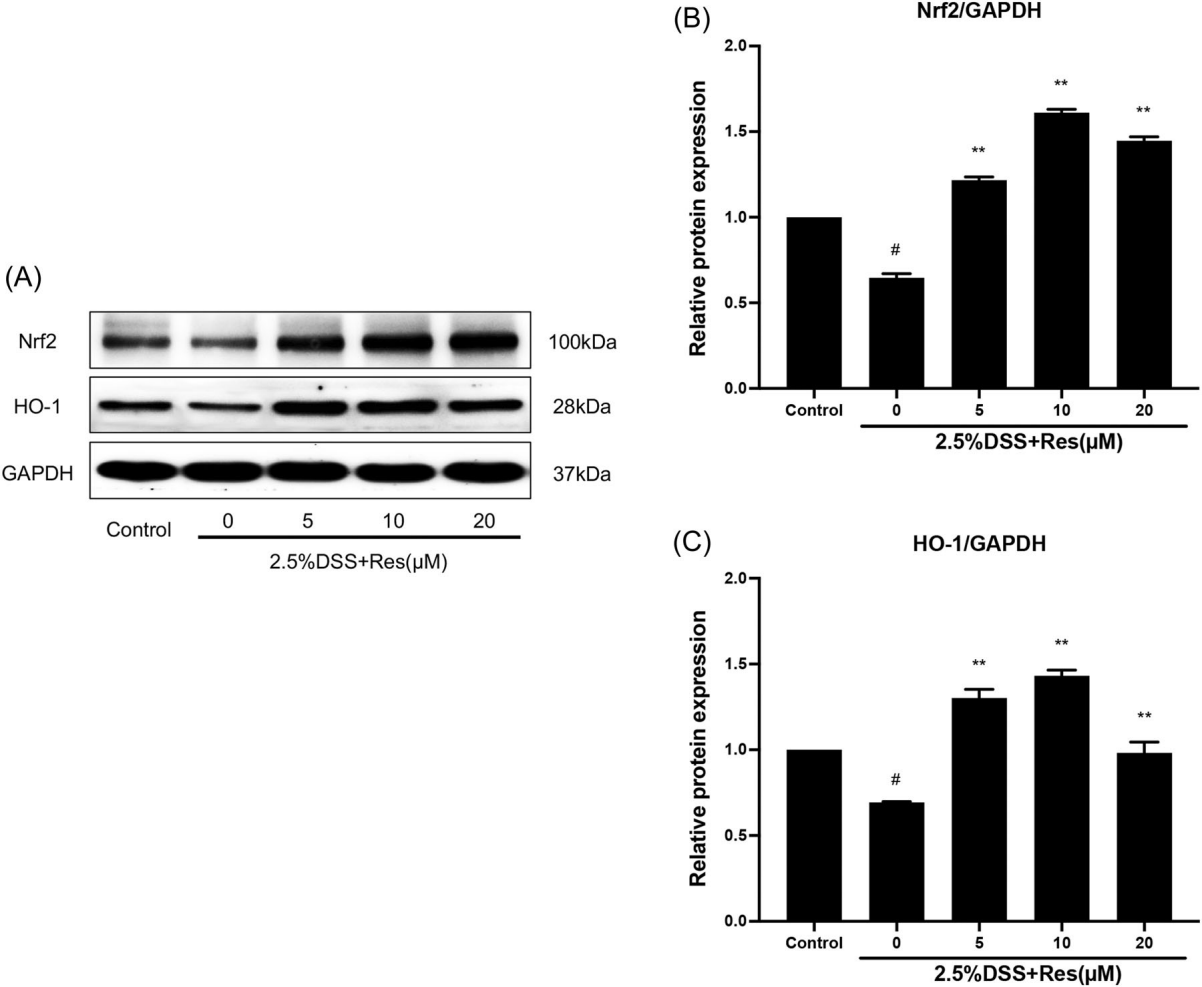
Resveratrol (Res) increases the expression of nuclear factor erythroid‐2‐related factor 2 (Nrf2) and heme oxygenase 1 (HO‐1). (A–C) Respectively Western blot and quantification data of Nrf2 and HO‐1. Intestinal epithelial cells (IECs) were pretreated with the indicated concentrations of Res for 1 h and then stimulated with 2.5% dextran sulfate sodium (DSS) for 24 h. ^#^ *p* < .01 versus control group; **p < .01 versus DSS group.

Activation of Nrf2 can suppress the inflammatory response and oxidative stress. Our investigation into Nrf2 and HO‐1 protein levels using Western blot analysis revealed an increase in their expression following Res pretreatment in DSS‐induced conditions (Figure 4A–C). These findings indicated that Res could activate Nrf2/HO‐1 pathway, thereby exhibiting its anti‐inflammatory and antioxidant properties. At the same time, we found that the expression of Nrf2/HO‐1 in each group was consistent with that of ZO‐1 and Occludin, that was to say, it was positively correlated with the expression of TJ proteins, which laid the foundation for our next verification.

### 3.5 Res upregulates Nrf2 and HO‐1 to protect IECs

**Figure 5:**
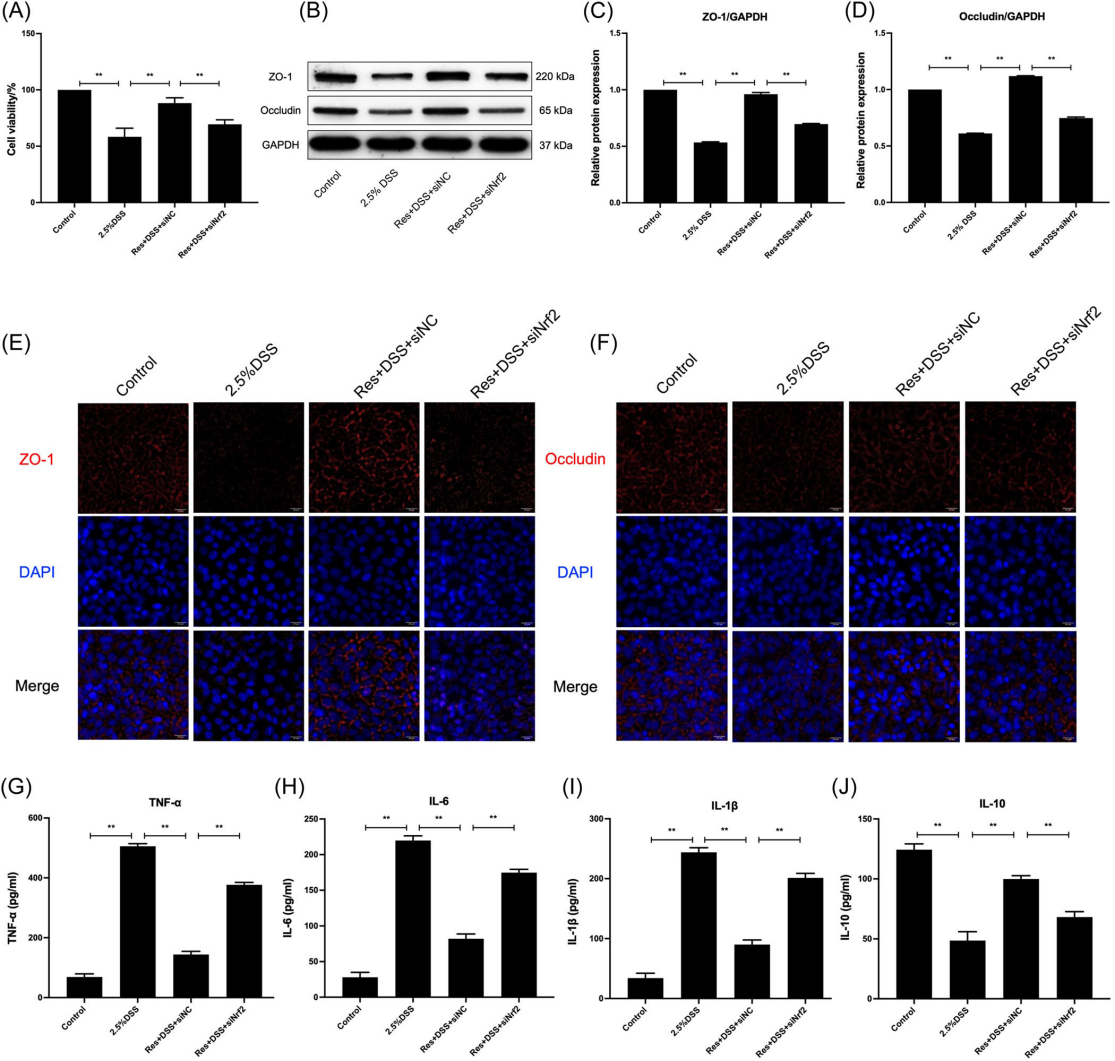
Nuclear factor erythroid‐2‐related factor 2/heme oxygenase 1 (Nrf2/HO‐1) pathway participates in the regulation of cell viability, tight junction (TJ) proteins, and inflammatory cytokines by resveratrol (20 µM). (A) Intestinal epithelial cells viability after Nrf2 knockdown. (B–D) The expression of TJ proteins (zonula occludens [ZO]‐1 and Occludin) after Nrf2 knockdown and subjected to Western blot. (E and F) ZO‐1 and Occludin distribution were assessed by immunofluorescence after Nrf2 knockdown. Scale bar, 20 µM. (G–J) Proinflammatory cytokines (tumor necrotic [TNF]‐α, interleukin [IL]‐6, and IL‐1β) and anti‐inflammatory cytokine (IL‐10) expression after Nrf2 knockdown were determined by enzyme‐linked immunosorbent assay. **p < .01.

The protective effect of Res (20 µM) on cell viability was notably diminished following Nrf2 knockdown (Figure 5A). Similarly, the expression of TJ proteins (ZO‐1 and Occludin) was significantly reduced in the Nrf2 siRNA transfection group compared to the siNC group (Figure 5B–D). Immunofluorescence analysis for ZO‐1 and Occludin aligned with these Western blot results (Figure 5E,F). Additionally, the protein expression levels of TNF‐α, IL‐6, IL‐1β, and IL‐10 were measured, revealing that Nrf2 knockdown negated the Res‐mediated anti‐inflammatory activity (Figure 5G–J). In summary, our findings suggested that Res upregulated Nrf2/HO‐1 pathway to play a protective role on DSS‐induced IECs.

## 4 DISCUSSION

**Figure 6:**
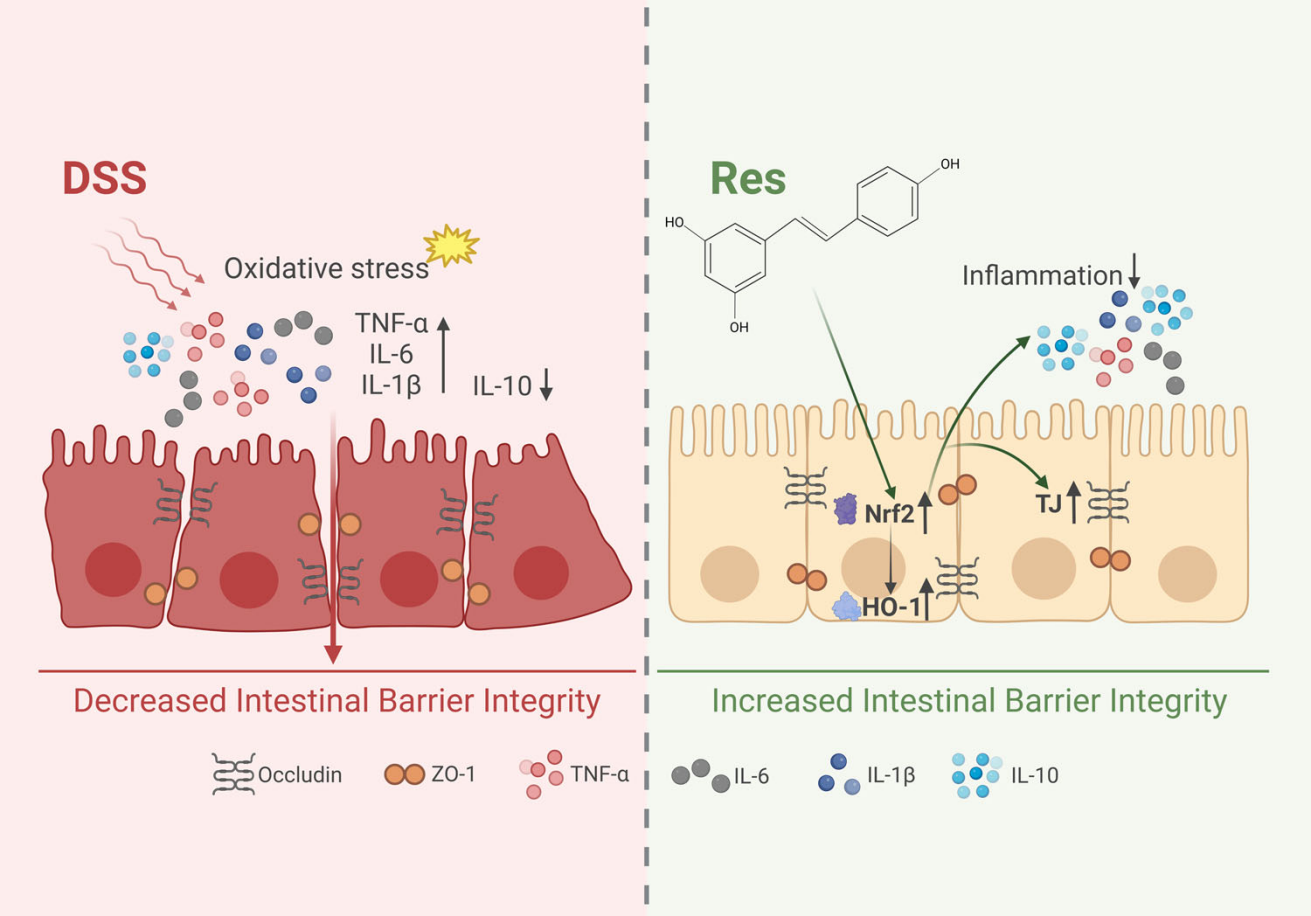
A model illustration of resveratrol activates nuclear factor erythroid‐2‐related factor 2/heme oxygenase 1 (Nrf2/HO‐1) pathway and attenuates dextran sulfate sodium (DSS)‐induced intestinal epithelial cells barrier dysfunction. Created with BioRender.com.

Maintaining intestinal homeostasis is crucial for human health. Disruptions caused by factors such as dietary habits, physical conditions, chemical substances, environmental influences, and genetic susceptibility can disturb the equilibrium between mucosal immunity and intestinal microbiota. These disturbances result in IECs barrier dysfunction, contributing to the development of various diseases.^29^ Numerous plant polyphenols have been recognized for their role in regulating intestinal health by improving intestinal morphology, digestion, absorption, immunity, and flora structure, and enhancing antioxidant and barrier functions.^30^, ^31^, ^32^ As a type of plant‐derived polyphenol, Res possesses a wide array of biological and pharmacological properties. However, the concentrations of Res in target tissues are typically low, rarely reaching pharmacological levels observed in in vitro studies.^33^ Despite ongoing debate regarding Res's efficacy, this study hypothesized that Res played a protective role in the IECs barrier dysfunction model induced by DSS, a well‐established model simulating the early inflammatory response of colitis.^34^ We observed that the administration of Res effectively attenuated DSS‐induced IECs barrier dysfunction and the protective effect was associated with anti‐inflammatory, antioxidant, increasing TJ proteins expression and activating the Nrf2/HO‐1 pathway (Figure 6), indicating that Res might be a potent, safe, and economical option for the treatment of intestinal diseases.

Growing evidence suggests that inflammatory responses stem from an imbalance between proinflammatory and anti‐inflammatory cytokines.^35^ The preliminary results of our study revealed that Res significantly reduced TNF‐α, IL‐6, and IL‐1β protein expression and increased that of IL‐10 (Figure 1). These results reflect those of Zhu et al. who also found that Res downregulated the expression of TNF‐α, interferon (IFN)‐γ, IL‐1β, IL‐6, and IL‐4 at the protein level in DSS‐induced acute UC mice.^36^ There are also similarities between the current findings and another study, which illustrated that Res ameliorated the production of inflammatory cytokines.^37^ Further in line with our findings, another study illustrated that the expression of IL‐10 is increased after γ‐Res treatment in inflammatory bowel disease.^38^ Thus, Res was able to regulate inflammatory cytokines production and exhibited a great potential to resist inflammatory response in colitis.

The integrity of the intestinal function is paramount. A decrease in TEER value in IECs monolayer compromises intestinal barrier integrity, elevating the risk of exposure to exogenous pathogenic factors.^39^ Our study used TEER measurement to assess Res's effect on IECs barrier integrity. While no significant change was observed in the control group, the resistance in the DSS‐induced cell model decreased markedly. However, Res pretreatment significantly reversed this phenomenon. Our result was consistent with previous studies showing the promotive effect of Res on the TEER no matter Caco‐2 cells or porcine intestinal IPEC‐J2 cells.^9^, ^40^ While the precise molecular mechanisms behind Res‐mediated improvement of IECs barrier integrity are unclear, it appears that the assembly of TJs contributes to the primary increase in TEER.^40^ TJs are key determinants of IECs barrier function and their disruption, leading to increased intestinal paracellular permeability, facilitates the infiltration of proinflammatory molecules into the intestinal tract and disrupts the mucosal immune system, resulting in intestinal inflammation.^41^ In recent years, Res has been shown to reduce intestinal inflammation, intestinal mucosal barrier dysfunction, and the histological score of colonic mucosal injury.^37^, ^42^, ^43^ Consistent with these studies, our results also show that the two critical TJ proteins ZO‐1 and Occludin expression and their distribution were significantly diminished following DSS treatment, but Res was able to protect both the key TJ proteins in a dose‐dependent mode (Figure 2). Hence, we demonstrated that Res could alleviate DSS‐induced IECs barrier dysfunction by enhancing its integrity and TJ proteins expression.

Res could relieve colitis and intestinal barrier dysfunction in multiple ways, for example, modulating gut microflora, downregulating microRNA‐31 to promote T regulatory cells, and mediating Wnt/β‐catenin pathway.^28^, ^44^, ^45^ Previous studies based on network pharmacology of Res have revealed that PI3K/Akt is the primary regulatory target of it, whether for UC or neurodegenerative diseases.^36^, ^46^ However, we identified a significant target, NFE2L2, also known as Nrf2, which was selected for further investigation (Figure 3). Nrf2, a transcription factor that regulates antioxidant and phase 2 detoxifying enzymes and associated proteins, serves as a vital sensor of oxidative stress in cells.^47^ Excessive ROS, a result of oxidative stress, can disrupt cellular signaling and is a crucial factor in IECs barrier dysfunction in intestinal diseases.^48^ As a downstream target protein of Nrf2, HO‐1, in conjunction with Nrf2, forms the Nrf2/HO‐1 pathway. This pathway is involved in the regulation and release of inflammatory cytokines and antioxidant factors, thereby influencing the development of colitis.^49^ Studies have shown that many plant polyphenols extracts, such as*Ligustrum lucidum*, *Salvia miltiorrhiza*, and aloe polysaccharides, could relieve symptoms of colitis by activating Nrf2/HO‐1 pathway, like diarrhea and bloody stool.^50^, ^51^, ^52^ Building on previous research, we observed that Res pretreatment markedly increased the expression of Nrf2 and HO‐1, indicating that Res exerts significant antioxidant activity by improving oxidative status (Figure 4). And the level of Nrf2/HO‐1 was negatively correlated with proinflammatory cytokines, and positively correlated with anti‐inflammatory factor and TJ proteins, so in the following experiment, we chose to inhibit the expression of Nrf2 rather than overexpress it. However, Nrf2 knockdown negated the protective effect of Res on cell viability, the enhancement of TJ proteins expression, and the regulation of inflammatory cytokines (Figure 5), implying that Nrf2 might be a principal target of Res in alleviating IECs barrier dysfunction, aligning with the predictions of network pharmacology analysis and validated our hypothesis.

## 5 CONCLUSIONS

Collectively, we investigated the protective role of Res on intestinal barrier function, highlighting its anti‐inflammatory and antioxidant properties in regulating key cytokine expressions. Additionally, we observed that Res pretreatment significantly activated the Nrf2/HO‐1 pathway in response to DSS‐induced IECs injury. The DSS‐induced IECs model served to investigate the initial interactions between DSS and IECs, mirroring the early stages of colitis in patients.^53^ In our study, DSS exposure led to cytotoxicity, barrier damage, inflammation, and oxidative stress, while Res countered these dysfunctions and the protective effects were achieved at least in part by upregulating the expression of Nrf2/HO‐1. Although this in vitro experiment cannot fully replicate real in vivo conditions, it underscored the protective effect of Res in the early stages of DSS‐induced colitis and provided a scientific foundation for subsequent in vivo studies. Overall, these findings may contribute to the establishment of Res as a therapeutic strategy for treating disorders associated with intestinal barrier dysfunction in colitis patients.

## AUTHOR CONTRIBUTIONS

**Xinya Yu**: Conceptualization; methodology; investigation; writing—original draft. **Yazhi Wang**: Conceptualization; methodology. **Yunchun Xu**: Investigation; formal analysis. **Xiaoxi Li**: Software; formal analysis. **Junhua Zhang**: Software; formal analysis. **Yunpeng Su**: Supervision; writing—review and editing. **Le Guo**: Conceptualization; supervision; project administration; funding acquisition; writing—review and editing. All authors have read and approved the final manuscript.

## CONFLICT OF INTEREST STATEMENT

The authors declare no conflict of interest.

## Data Availability

The data used to support the findings of this study are included within the article.
